# Generating Insights from Trends in Newborn Care Practices from Prospective Population-Based Studies: Examples from India, Bangladesh and Nepal

**DOI:** 10.1371/journal.pone.0127893

**Published:** 2015-07-15

**Authors:** Sonya Crowe, Audrey Prost, Munir Hossen, Kishwar Azad, Abdul Kuddus, Swati Roy, Nirmala Nair, Prasanta Tripathy, Naomi Saville, Aman Sen, Catherine Sikorski, Dharma Manandhar, Anthony Costello, Christina Pagel

**Affiliations:** 1 Clinical Operational Research Unit, University College London, 4 Taviton Street, London, WC1H 0BT, United Kingdom; 2 Institute for Global Health, University College London, 30 Guilford Street, London WC1N 1EH, United Kingdom; 3 Perinatal Care Project, Diabetic Association of Bangladesh, 122 Kazi Nazrul Islam Avenue, Dhaka 1000, Bangladesh, India; 4 Ekjut, Plot 556B, Potka, Chakradharpur, West Singhbhum, Jharkhand, India; 5 Mother Infant Research Activities (MIRA), YB Bhavan, Thapathali, GPO Box 921, Kathmandu, Nepal; London School of Economics, UNITED KINGDOM

## Abstract

**Background:**

Delivery of essential newborn care is key to reducing neonatal mortality rates, yet coverage of protective birth practices remains incomplete and variable, with or without skilled attendance. Evidence of changes over time in newborn care provision, disaggregated by care practice and delivery type, can be used by policymakers to review efforts to reduce mortality. We examine such trends in four areas using control arm trial data.

**Methods and Findings:**

We analysed data from the control arms of cluster randomised controlled trials in Bangladesh (27 553 births), eastern India (8 939), Dhanusha, Nepal (15 344) and Makwanpur, Nepal (6 765) over the period 2001–2011. For each trial, we calculated the observed proportion of attended births and the coverage of WHO essential newborn care practices by year, adjusted for clustering and stratification. To explore factors contributing to the observed trends, we then analysed expected trends due only to observed shifts in birth attendance, accounted for stratification, delivery type and statistically significant interaction terms, and examined disaggregated trends in care practice coverage by delivery type. Attended births increased over the study periods in all areas from very low rates, reaching a maximum of only 30% of deliveries. Newborn care practice trends showed marked heterogeneity within and between areas. Adjustment for stratification, birth attendance and interaction revealed that care practices could change in opposite directions over time and/or between delivery types – e.g. in Bangladesh hygienic cord-cutting and skin-to-skin contact fell in attended deliveries but not home deliveries, whereas in India birth attendant hand-washing rose for institutional deliveries but fell for home deliveries.

**Conclusions:**

Coverage of many essential newborn care practices is improving, albeit slowly and unevenly across sites and delivery type. Time trend analyses of birth patterns and essential newborn care practices can inform policy-makers about effective intervention strategies.

The global burden of neonatal and maternal deaths is high in South Asia, with approximately 1.2 million newborn deaths and 83 000 maternal deaths each year [1,2]. Although neonatal mortality in this region has been falling, significant improvements in essential newborn care remain a priority [3]. Policy has focused mainly on increasing access to skilled attendance at birth, both by raising the level of institutional deliveries and increasing access to a skilled birth attendant (SBA) for home deliveries [4–6]. There have also been efforts to improve aspects of newborn care for women giving birth at home without an SBA, for example through the promotion of clean delivery kits [7,8]. However the coverage of protective birth practices remains incomplete and variable across settings [9–15]. Observed improvement in the coverage of essential newborn care could be due to more women having a skilled attendant at delivery, improvements in the quality of skilled attendance, improvement in practices at unattended deliveries at home or a combination of all three. Understanding the balance of reasons for observed trends would greatly enhance the information available to policy-makers.

Over the last decade, community trials aimed at testing interventions to improve maternal and neonatal health have generated detailed information about birthing care practices in rural South Asia. In a recent publication [15], we used information from the control arms of four cluster randomised controlled trials conducted in rural areas of Nepal, Bangladesh and India [16–20] to describe the coverage of newborn care practices for births at home and within health facilities in these areas. This revealed shortfalls in the coverage of newborn care that varied markedly between geographical areas and according to attendance at birth. This motivated the focus of this work, namely to explore how the provision of care was changing over time in these areas.

Evidence of changes over time in the provision of newborn care at attended and unattended deliveries can usefully inform policy, and indeed are probably most useful when used by local decision-makers who have a detailed understanding of the local context and initiatives to improve care. Disaggregated trends in essential newborn care by care practice, place and type of delivery can provide a valuable means of gauging the impact of past and ongoing initiatives and be used to identify areas where least progress has been made so that future interventions can be effectively prioritised. An example of the use of such information is the analysis of trends in child mortality in four Indian states by Nguyen et al [21] and Minnery et al. [22] disaggregated across a range of markers in order to better understand the impact of previous policy initiatives and the current priorities. Similarly, Acuin *et al*. used time trend analysis of maternal, neonatal and child health in southeast Asia to look for factors that might accelerate progress and pose questions for future policy [23].

Our aim in this paper was to understand recent trends in birth care practices from four cluster randomised trials (in Nepal, Bangladesh and India) from control arm data. We provide interpretations of these analyses for each site in order to illustrate the insights that can be gained through this approach. We do not attempt (or intend this analysis to be used for) direct comparisons across the four trials.

For each trial, we assessed how access to skilled attendance at birth (whether at home or in an institution) changed over time, and how this related to trends in the coverage of different newborn care practices. We then compared trends in the care provided at attended and unattended births, and considered underlying factors contributing to the changes observed. Specifically, we asked three research questions:

Q1. What was the observed trend in birth attendance?

Q2. What were the observed trends in the coverage of birthing care practices?

Q3. What factors contribute to the observed trends in care practices?

In addressing these questions for each trial, we aimed to provide examples of how evidence can support local or national policy-makers in developing strategies for improving health care provision for mothers and newborn infants. While the specific data shown are most relevant to the regions where the four trials took place, our approach could be usefully adopted more widely where control arm trial data on community practices exists. Whilst we acknowledge that different trials are not necessarily directly comparable, this type of analysis published alongside the main trial results for a given site could provide policy-makers and researchers with valuable insights.

## Methods

### Study population

The data used in this study originated from four cluster randomised controlled trials in rural settings in eastern India, Bangladesh and Nepal. The eastern Indian trial was based in three districts of Jharkhand and Odisha (Keonjhar, West Singhbhum and Saraikela) [20]. The Bangladesh trial included the districts of Bogra, Maulvibazaar and Faridpur [18,19]. The Nepal trials were based in the middle hills district of Makwanpur [16], and the plains district of Dhanusha [17]. The interventions in all of these trials involved four-phase participatory learning and action cycles with women’s groups, details of which are presented elsewhere [16–20]. Key characteristics of each trial are summarised in S1 Table.

The trials attempted to identify and enrol all women who gave birth during the trial period, except for the Dhanusha trial (Nepal), which sampled births (10 per cluster per month), and the Makwanpur trial (Nepal) in which pregnancies (rather than deliveries) were identified. The estimated study populations ranged from 228 000 (Eastern India) to 670 000 (Dhanusha, Nepal). Detailed questionnaires were used to collect information regarding the intrapartum care of all enrolled women. In this study, we use information regarding newborn care practices gathered in the control clusters of the trials to examine how these practices changed over time in the absence of the trial interventions; we were not evaluating the trial interventions, so did not use data from the women’s group intervention clusters [15].

### Study periods

**Fig 1:**
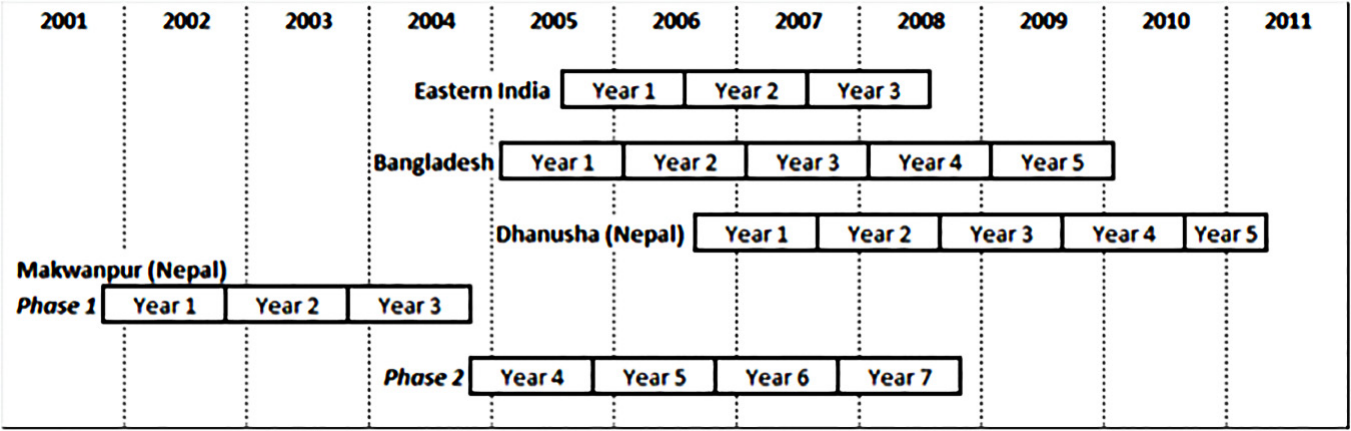
Analysis study periods. The study periods used in the analysis, for each site. “Year” refers to the study year, i.e. the 12-month period from the start of the study (see text).

Fig 1 summarises the time periods covered in this study. We note that some trials started and/or ended part way through a Gregorian calendar year and that we used surveillance data from outside the defined trial periods for some study areas. In our analysis we make an important distinction between calendar years and study years, which we define as sequential 12-month periods from the start of the study (with the exception of Dhanusha, which begins at the start of the surveillance period and has 8 months of data in study year 5). We use Gregorian calendar years to compare trends in birth attendance across all sites and study years when examining changes in care practice coverage in individual sites. The Makwanpur (Nepal) site comprises two study phases. Initially there were 12 non-women’s group control clusters in the trial (Phase 1). After three years, women’s groups were introduced in these control clusters and six new non-women’s group control clusters were recruited (Phase 2).

### Defining care practices and birth type

The World Health Organization (WHO) Guide to Essential Newborn Care [24] was used to identify and match newborn best care practices with data collected during each trial, using the closest match to the WHO definition where there was no exact match [15]. The WHO recommendation and detailed questions asked for each practice in each trial are given in S2 Table. The care practices identified by the WHO are based on best available evidence and represent “a common understanding between WHO, UNFPA, UNICEF, and the World Bank of key elements of an approach to reducing maternal and perinatal mortality and morbidity” [24]. The WHO recommends several practices related to thermal care. We use the following range of thermal care practices in our analyses (where available), all of which are known to protect newborn infants from hypothermia in the immediate post-delivery period [25]: skin-to-skin contact within 30 minutes; immediate wiping (within 10 minutes); delayed bathing (not within 6 hours for Bangladesh, eastern India and Makwanpur, not within 24 hours for Dhanusha); immediate warmth (wrapping within 5 minutes for Dhanusha, wrapping within 10 minutes for Makwanpur and wrapping *or* skin-to-skin contact within 10 minutes for eastern India and Bangladesh).

The full list of identified intrapartum care practices is given in S3 Table along with a description of each practice. Note that only the Nepal studies had specific data on colostrum and only the eastern India and Bangladesh studies had data regarding use of a plastic sheet, gloves, boiled thread to tie the cord, a clean cloth for wrapping, skin-to-skin contact, immediate wiping and giving only breast milk in the first day. Makwanpur, Nepal did not have data regarding thread/clamp use in Phase 1 and neither Nepal study had data regarding whether a *new* blade specifically was used to cut the cord but instead asked about whether the blade was boiled or not.

Each included birth was defined as either “attended” (if it took place in an institution or at home with a skilled birth attendant (SBA) or “unattended” (if it took place at home without an SBA). An institution was defined as a private hospital, health post, primary health care centre, government health centre, charitable hospital or a maternal and child welfare centre. Doctors, nurses and auxiliary nurse midwifes were defined as SBAs in all study areas. In the eastern India and Bangladesh studies government health workers were also defined as SBAs. An SBA was required to be the main attendant in a non-institutional birth for the birth to qualify as “attended”. Consistent with terms used by the World Health Organization [26], traditional birth attendants (TBAs) were not considered as skilled birth attendants. We note that 15% of all deliveries in the control arm in Bangladesh were by TBAs given four days of training in safer care practices for a trial testing use of bag and mask for neonatal resuscitation [18,27]. A detailed analysis of newborn care practices disaggregated by TBA attendance is given in Pagel *et al*. [15]. The exact definitions used for each delivery type in each study area are given in S4 Table. Births for which the mother was recorded to have transferred to an institution during delivery were defined as “attended” for the post-delivery care practices.

The decision to combine institutional and home SBA deliveries into a single birth type (“attended”) was partially driven by the need for sufficient numbers to conduct statistical analysis, and also reflects our previous work that markedly better coverage of newborn care practices are observed in both cases compared to home births without an SBA [15].

### Data exclusions

Data where the type of the birth (attended/unattended) could not be determined were excluded. We did not include records for which the mother migrated out of the study area or was lost to follow-up and records where the mother or infant died in the antenatal period. All multiple births except for the first born child were excluded to avoid counting practices for the same delivery multiple times. For each newborn care practice, records where that care practice information was missing were excluded from that particular analysis. For post-delivery newborn care practices, we also excluded stillbirths and intrapartum maternal deaths since these events could have changed the course of the delivery. Following data exclusions, there were records for 8 939 births for eastern India, 27 553 births for Bangladesh, 6 765 births for Makwanpur and 15 344 births for Dhanusha.

### Data analysis

**Fig 2:**
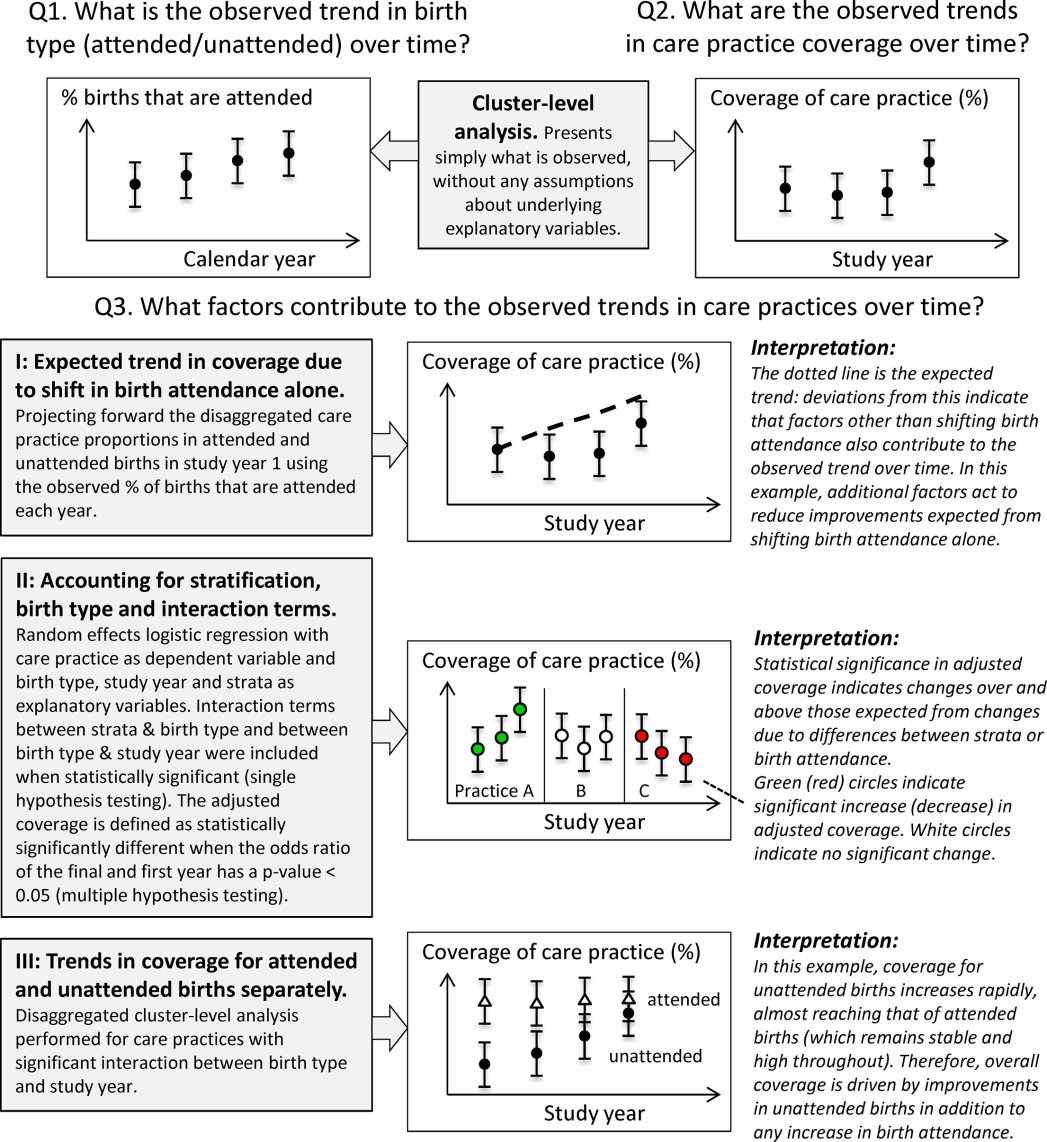
Schematic diagram of analysis. The analysis conducted to address the three key research questions posed is shown schematically.

An outline of the analysis conducted to address, for each study, the three key research questions posed in the introduction is shown schematically in Fig 2. The underlying contributing factors to the observed trends in care practice coverage (research question 3) are complex and so we present a range of analyses.

#### Observed trends in birth attendance over time (Research question 1)

The observed proportion of attended births was calculated for each trial site by calendar year, along with 95% confidence intervals, using the cluster-level analysis methods set out in Hayes [28] and accounting for stratification by specific geographical areas in the eastern India, Bangladesh and Dhanusha trials. Calendar year was chosen as the time unit to facilitate comparison across sites for this research question (in contrast to the other research questions for which *study* year is used).

#### Observed trends in coverage of care practices over time (Research question 2)

We defined the coverage of a given care practice as the percentage of births for which that practice was recorded as having occurred. The cluster-averaged coverage of each care practice was calculated for each study year at every site, along with 95% confidence intervals, adjusting for stratification where appropriate (eastern India, Bangladesh, Dhanusha) [28]. This cluster-level analysis has the advantage of being robust across all combinations of study year and care practice, providing a consistent method across all studies. It is also an effective means of presenting what is observed without any assumptions about underlying explanatory variables. However, the confidence intervals are large for these point estimates and individual-level analysis provides complementary information (see below).

### Factors contributing to observed trends in care practices (Research question 3)

We used three complementary analyses to examine this question from different perspectives.

1. Expected trends due only to observed shift in birth attendance: For every study year of each study, we calculated the expected *overall* coverage of each care practice associated with the observed shift in the proportion of attended births over the study period, assuming that the coverage *within* attended or unattended births remained unchanged from study year 1. For example, consider a study area in which 10% of births were attended in year 1, 20% in year 2 and 30% in year 3. Let us also imagine that in year 1 of the study, coverage of care practice X is 80% for attended births and 50% for unattended births. In year 1, the overall coverage of care practice X would be 53% (10% x 80% + 90% x 50%). Now let us assume that the only change over time is the increase in birth attendance and hence the coverage of care practice X for attended and unattended births remains 80% and 50% respectively. The expected overall care practice coverage due to the shift in birth attendance alone may then be calculated for year 2 as 20% x 80% + 80% x 50% = 56%. Similarly, we calculate this for year 3 as 30% x 80% + 70% x 50% = 59%. Deviations of the observed coverage from the expected trend are used as a guide to indicate where factors other than changes in birth attendance also contribute to the observed coverage (e.g. local or national policy initiatives).
  Note that for Makwanpur and Dhanusha, some care practices had very few responses for attended deliveries in some or all clusters resulting in misleading coverage for study year 1. Therefore we do not present expected trends for care practices where there were fewer than ten responses in two or more clusters.
2. Accounting for stratification, birth type and interaction terms: There is evidence of differences between the coverage of care practices in attended and unattended births [15]. Coverage may also differ between geographic strata. In order to take these factors into account in interpreting the observed changes in care practice coverage over time, we performed random effects logistic regressions in which the care practice was the dependent variable and birth type, study year and geographic stratum (eastern India, Bangladesh, Dhanusha) were treated as explanatory variables along with their interactions. A Bonferroni correction was used to account for multiple hypothesis testing across care practices for a given study. A quadrature check was performed for all combinations of care practice and study year, for each study area, to check for reliability of the model fit.
  Logistic regression odds ratios between the first and final study year with a p-value < 0.05 after the Bonferroni correction were interpreted as evidence of changes in care practice coverage over and above those resulting from differences between geographic strata or changes in where women give birth: we define these as statistically significant changes in *adjusted* care practice coverage over time. Odds ratios less (greater) than 1 are interpreted as a greater decrease (increase) in coverage than expected from changes in included explanatory variables alone. We note that, since we are not testing a hypothesis or intervention, the odds ratios themselves are not presented and the significance tests should be viewed by readers as a guide to interpreting whether the observed trends can be explained by secular increases in delivery type or differences between regions.
3. Trends in care practice coverage by birth type over time: For care practices showing significant changes in adjusted care practice coverage over time, we explore whether these changes are similar between attended and unattended births (since this might provide useful information for policy) by visually comparing trends over time for each care practice disaggregated by birth type. To reduce the number of plots, we look only at care practices where the interaction between study year and birth type in the regression (method *II*) was significant (accounting for multiple hypothesis testing using a Bonferroni correction). For these care practices, the cluster-averaged coverage within attended and unattended births was calculated for every year of each study, along with 95% confidence intervals, adjusting for stratification where appropriate (eastern India, Bangladesh, Dhanusha). Some care practice / birth type combinations had very few responses in some or all clusters (particularly for attended deliveries), often attributable to a jump sequence in the questionnaires. We do not present estimates by birth type where there were fewer than ten responses in two or more clusters, since these estimates are unlikely to be meaningful.
  Stata/IC 12.1 (StataCorp LP) was used for all data analysis. We direct the reader to Fig 2 as a summary of the research method and ways of presenting results.

### Ethics

All trials from which data for this study were drawn were approved by the ethics committee of the Institute of Child Health and Great Ormond Street Hospital for Children (UK) and by the following research ethics committees: the ethical review committee of the Diabetic Association of Bangladesh; an independent ethics committee in Jamshedpur, India (Eastern India trial); the Nepal Health Research Council (Dhanusha and Makwanpur, Nepal). All trials were conducted in disadvantaged areas with high levels of female illiteracy; all participants gave consent in writing, by thumbprint or verbally.

## Results

### Observed trends in birth type over time (Research question 1)

**Fig 3:**
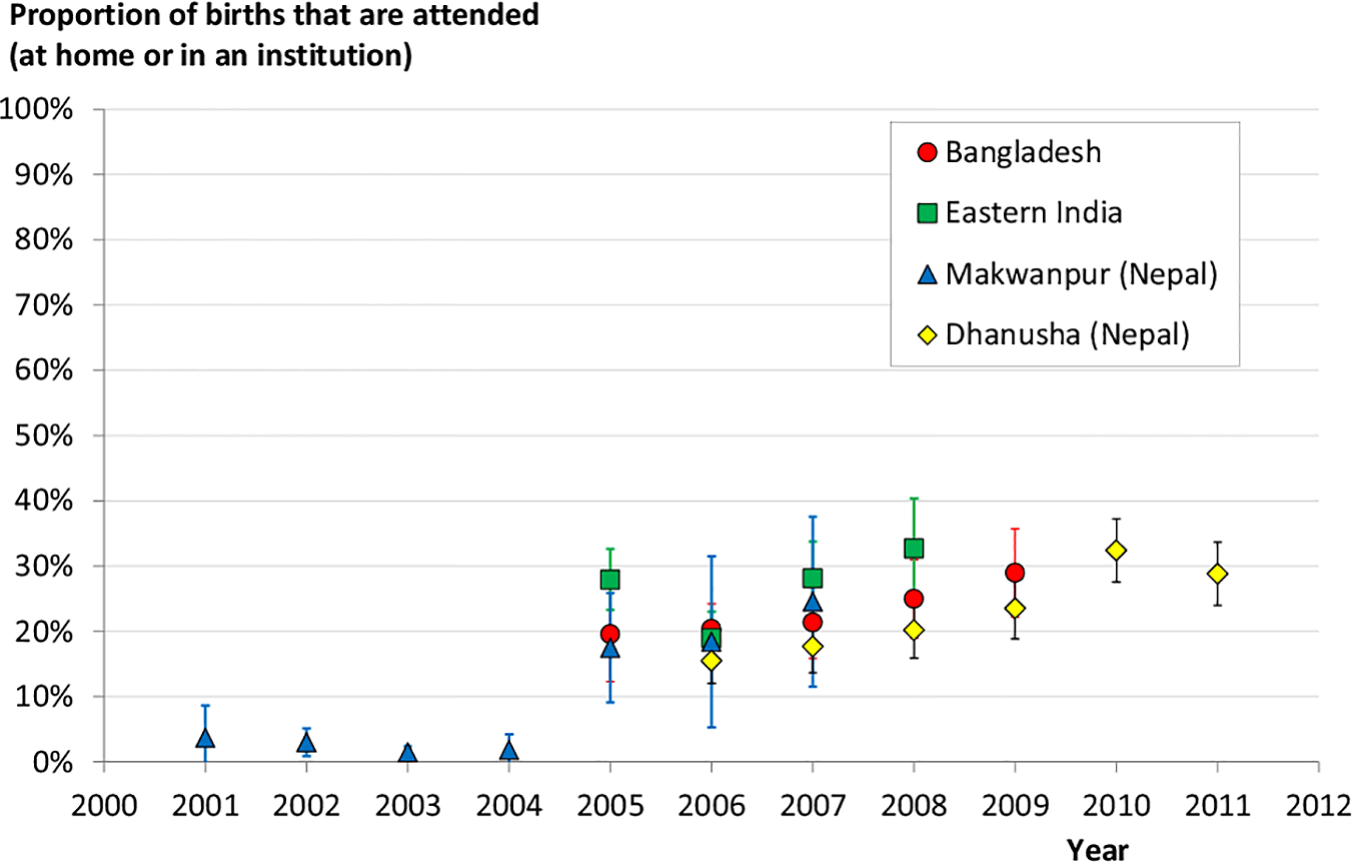
Trends in birth attendance. Proportion of births taking place in an institution or at home with a skilled birth attendant by year, in each study area (with 95% confidence intervals). Note that for Makwanpur there are very few observations in the control arm in 2008 and so the 95% confidence intervals become very large (2.9%, 68%), hence this data point was considered meaningless and is not shown. Note also that for eastern India, 2005 only contained five months of data including an anomalously high first month.

The change over time of the proportion of births taking place in an institution or at home with an SBA (i.e. “attended”) is shown in Fig 3 for each study area. The proportion of attended births increases over the study periods in all areas. However, all areas show relatively low rates of attended births and modest increases, with the most recent observations reaching only 30% of deliveries with skilled attendance.

### Observed trends in coverage of care practices over time (Research question 2)

**Fig 4:**
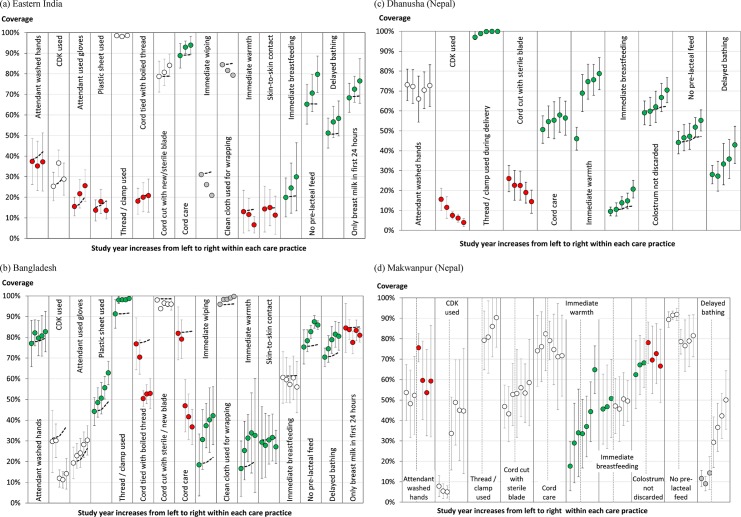
Trends in care practices. The cluster-averaged coverage of each care practice in each study year for (a) Eastern India, (b) Bangladesh, (c) Dhanusha (Nepal), and (d) Makwanpur (Nepal), with 95% confidence intervals. Study year increases from left to right within each care practice. The two phases of the Makwanpur study are separated by dotted vertical lines within each care practice. The dashed lines show the expected trend resulting from the observed shift in birth attendance alone, assuming the care practice coverage for attended and unattended births remain unchanged from study year 1 (see section *I)*. Green (red) circles denote care practices with a statistically significant increase (decrease) in *adjusted* coverage between the first and last study year as determined from logistic regression modelling (see section *III)*. Note that a significant change in *adjusted* coverage can be in the opposite direction to the trend observed in the cluster-adjusted coverage. White circles denote care practices with no statistically significant difference in coverage between the first and last study year after adjustment for geographic strata and birth attendance, whilst grey circles denote care practices for which the logistic regression quadrature check showed that the fit was not reliable.

Fig 4A–4D show the cluster-averaged coverage for each available birth practice in each year of the eastern India, Bangladesh, Dhanusha and Makwanpur studies respectively. Within each care practice, study year increases from left to right. Note that the study years correspond to different time periods in each of the studies, with Phase 1 of the Makwanpur trial occurring much earlier than the other trials. The dashed trend lines and colour coding of the data points relate to research question 3 (factors contributing to observed trends in care practices) and are explained in results sections *I* (expected trends due to observed shift in birth attendance alone) and *III* (trends in care practice coverage by birth type over time) below.

### Factors contributing to observed trends in care practices (Research question 3)

#### I. Expected trends due to observed shift in birth attendance alone

The dashed lines in Fig 4A–4D show the expected trend associated with the observed shift in the proportion of attended births over time (Fig 3) assuming that the coverage of care practices at attended or unattended births remained unchanged from study year 1. Many of the observed trends deviate from these expectations, suggesting that there are other important contributing factors that should be explored and that skilled birth attendance alone is not accounting for changes.

#### II. Accounting for stratification, birth type and interaction terms

In Fig 4A–4D, green (red) circles denote care practices with a statistically significant increase (decrease) in adjusted coverage in the first and last study year as determined from logistic regression modelling. Note that a significant change in adjusted coverage may be in the opposite direction to the trend observed in the unadjusted cluster-average coverage, as for instance with attendant glove use in eastern India (which significantly decreases after adjustment (red circles), despite the increasing cluster-average trend, see discussion below). White circles denote care practices with no statistically significant difference in coverage between the first and last study year after adjustment for geographic strata and birth attendance, and grey circles denote care practices for which the logistic regression quadrature check showed that the fit was not reliable and so no statement about significance can be made.

#### *III*. Trends in care practice coverage by birth type over time

**Fig 5:**
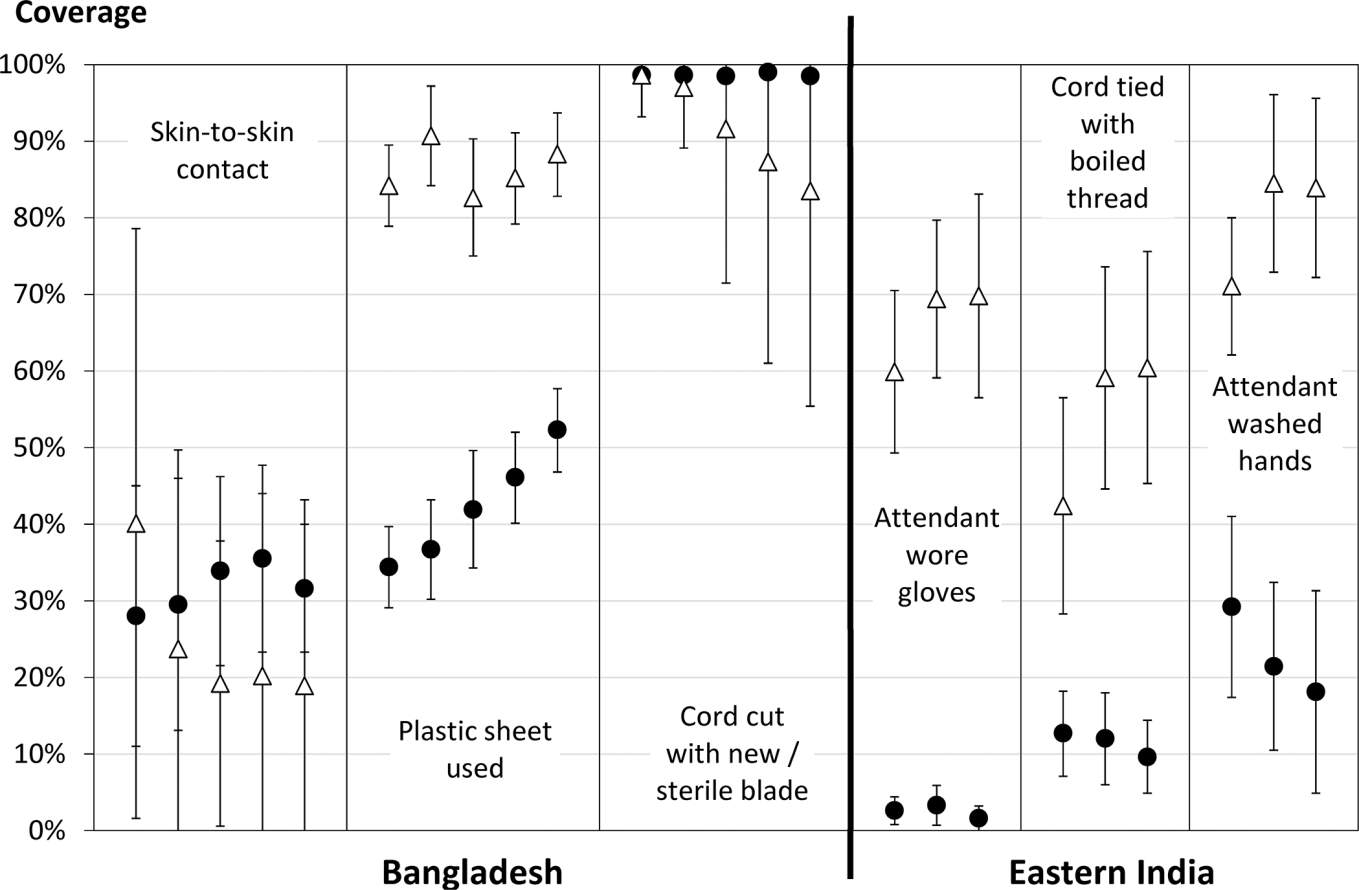
Disaggregated trends in care practices. Cluster-average coverage of attended (triangles) and unattended (circles) births for a given care practice in the Bangladesh (left) and eastern India (right) studies over time, with 95% confidence intervals. The study year increases from left to right within each care practice. Information is displayed only for those care practices for which there was a significant change in the overall adjusted coverage over time and a significant interaction term between birth type and study year (method *II*), and for which attended and unattended births displayed contrasting behaviour during the given study period.

Fig 5 shows the cluster-averaged coverage in attended (triangles) and unattended (circles) births for a selection of protective care practices during the eastern India and Bangladesh studies. We present only those care practices for which there was a significant change in adjusted coverage over time and significant interaction term between birth type and study year (method *II*) *and* for which attended and unattended births displayed contrasting behaviour during the given study period.

## Discussion

Illustrative application of these analyses for each study: what might a policy maker or researcher ask when looking at this data?

**Fig 6:**
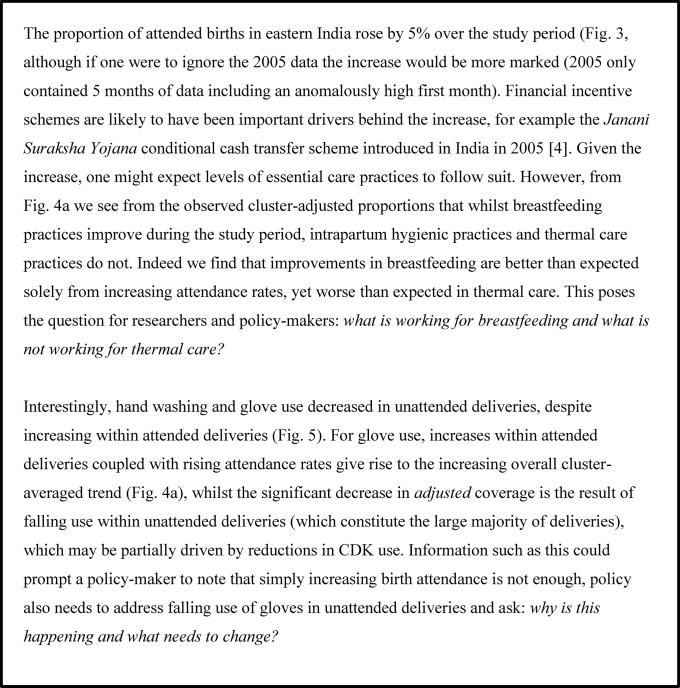
Illustrative application: Eastern India. Interesting analysis findings for Eastern India, highlighting the sorts of potential learning and questions for further inquiry that would be of most relevance to researchers and policy-makers in these areas.

**Fig 7:**
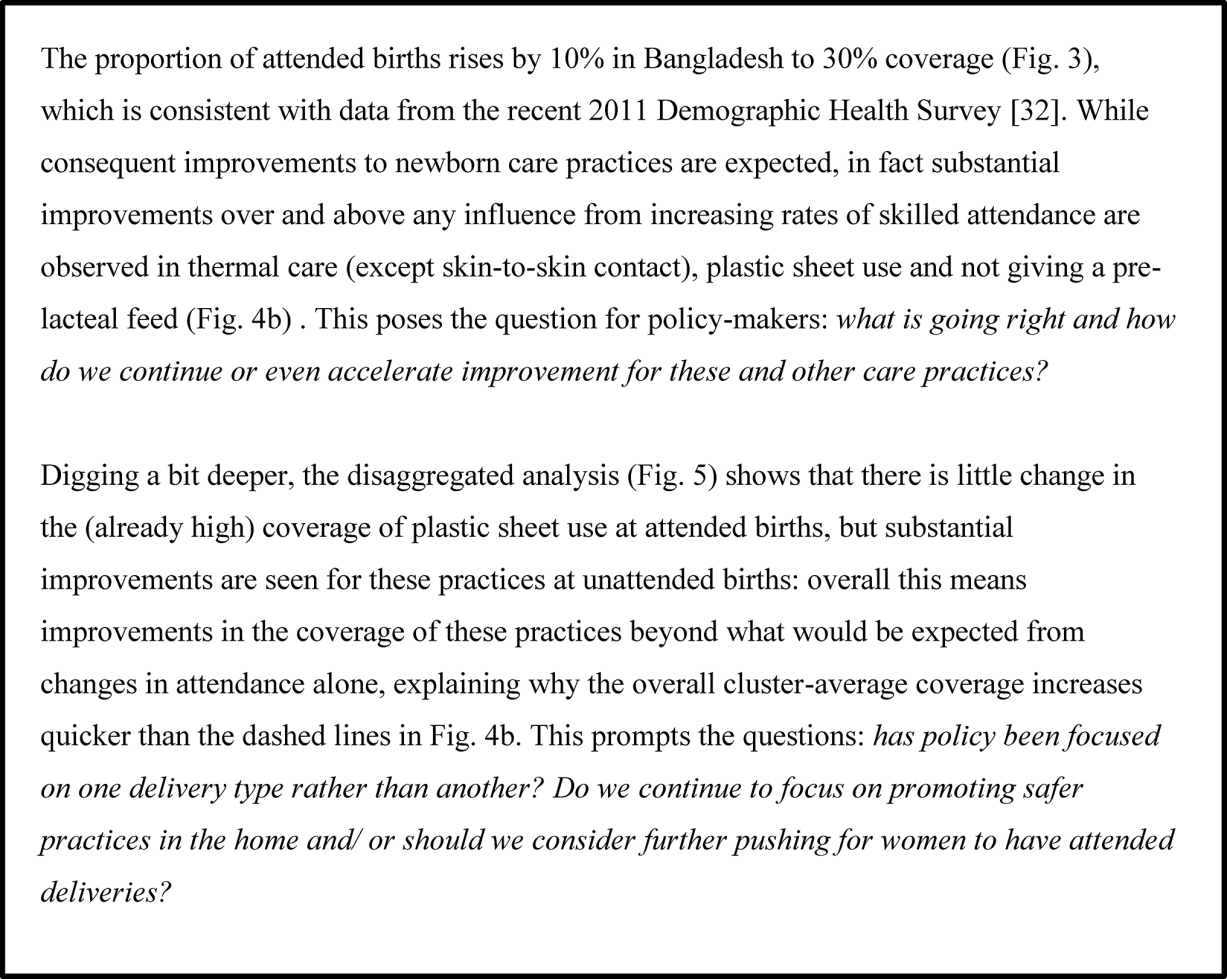
Illustrative application: Bangladesh. Interesting analysis findings for Bangladesh, highlighting the sorts of potential learning and questions for further inquiry that would be of most relevance to researchers and policy-makers in these areas.

**Fig 8:**
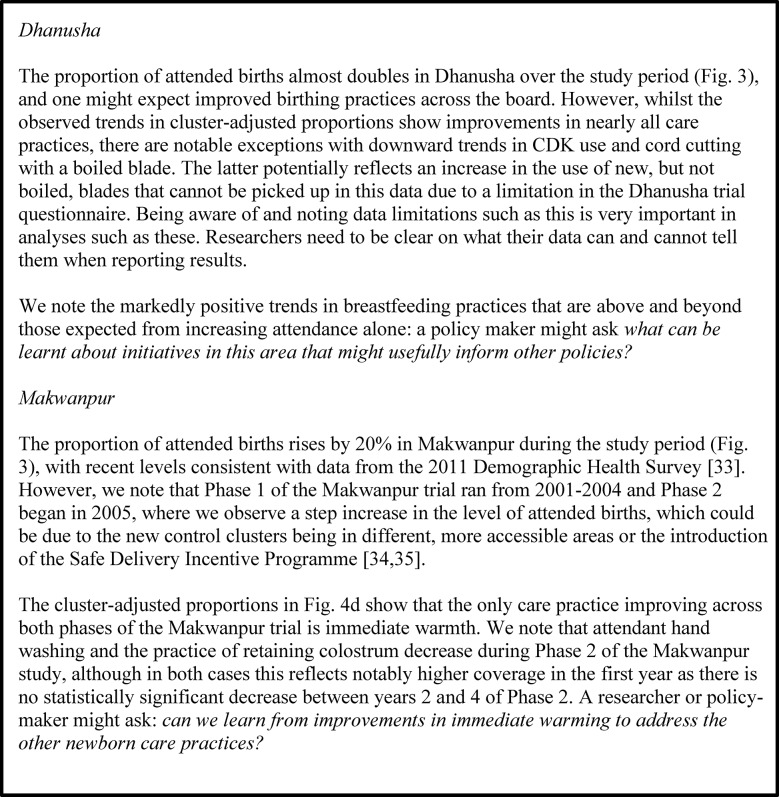
Illustrative application: Nepal. Interesting analysis findings for Dhanusha and Makwanpur, highlighting the sorts of potential learning and questions for further inquiry that would be of most relevance to researchers and policy-makers in these areas.

In Figs 6–8 we present an interpretation of interesting analysis findings for each study, highlighting the sorts of potential learning and questions for further inquiry that would be of most relevance to researchers and policy-makers in these areas. Since these are intended to demonstrate the utility of this sort of analysis rather than concentrate on the details of individual studies, we do not discuss every aspect of all analyses in each study. We note that a broader discussion of postnatal care practices across the four areas can be found in Pagel el al. [15].

### Key findings

Data originating from four cluster-randomised controlled trials based in rural areas of Nepal, Bangladesh and eastern India have been used to explore how changes over time in the provision of newborn care in the context of changing rates of deliveries with skilled attendance can provide valuable information that could support programme managers and policy-makers in developing future interventions. Whilst the four trials presented here present valuable information on over 58,000 births in rural, poor communities, they still pertain to relatively small areas in south Asia. The specific trends presented here will be different for other areas and countries and are also likely to be markedly different for urban rather than rural contexts. However, our work shows clearly that considering “essential newborn care” as a single package of care or equating attended births with “full coverage of essential newborn care” and unattended births with “no coverage of essential newborn care” loses important information. The coverage of different care practices can change in opposite directions over time and/or in opposite directions between attended and unattended deliveries. Observed changes in coverage of individual care practices can match what would be expected simply by changes in birth attendance, exceed expected increases, or not meet expected increases. These findings have two major implications. First, they demonstrate that a focus on quality of care across the continuum encompassing pregnancy, childbirth and the postnatal period is important, and duly recognized by the 2014 Every Newborn Action Plan [29]. Second, it will be beneficial to retain questions that enable prospective monitoring of the coverage of practices in national and district-level surveys, and not to assume that attended births have optimal coverage of all practices.

### Limitations of the study data

In using this kind of approach, it is of course important to also present the limitations in the data used. We give the main limitations of the study data used here below.

We cannot know the accuracy of a woman’s recollection and there may have been systematic differences between settings in how women responded to questions. Similarly, we cannot know the extent of interviewer bias (e.g. a tendency to ask questions or record answers differently from others) across the different settings. There may also be study-specific changes in reporting over time (e.g. with interview fatigue). We note that the recollection of events during deliveries in an institution may be biased by women assuming aspects of care were provided when they were not, although this bias is unlikely to change over time (and hence unlikely to affect trends). A limitation specific to the trend analysis of the Makwanpur study (Nepal) was the presence of different clusters in the two study phases, which therefore had to be analysed separately.

It was not always possible to separate trends in attended deliveries from trends in unattended deliveries due to a combination of relatively few home births attended by a skilled birth attendant within each cluster, which lead to appreciable variation between clusters, and high rates of missing data for attended deliveries (levels of which were variable between study areas). All of the newborn care practices in the Nepal studies (except the immediate breastfeeding, retaining colostrum and pre-lacteal feeding practices in Dhanusha), had too few responses (<10) within the attended birth category in some or all clusters to perform robust cluster-average analysis. In the case of Dhanusha, the missing data for attended deliveries was largely due to a ‘skip sequence’ in the questionnaire that resulted in the questions regarding thermal care and hygiene practices simply not being asked in institutional deliveries. We note that this effect increased after 2008/9 (affecting study years 3–5) when the questionnaire was slightly altered, which may skew the aggregated trend results presented in 4c. Thus the plots for attended care practices (other than breastfeeding) for Dhanusha should be treated with caution but they do illustrate the range of trends possible with these sorts of plots. We also note that the large majority of deliveries in Dhanusha are unattended home births (not affected by the jump sequence mentioned above) and thus observed trends in Fig 4C will be most influenced by these deliveries.

Many of the cluster-averaged estimates have large confidence intervals due to a combination of genuinely large variability in coverage and the effect of using cluster-level analysis techniques. This was partly addressed through the significance testing of the random effects logistic regression models, although we note that this analysis did not take into account many factors that may also contribute to the trends observed, such as changes in socio-economic status and maternal education.

### Implications

Despite increases in skilled attendance at birth, most South Asian women in rural areas still give birth at home without an attendant [30], so promoting appropriate care practices in these settings remains critical. Indeed, trends in overall coverage of a number of care practices were below expectations from shifting birth attendance alone, suggesting that efforts to increase attendance cannot be relied upon as a policy tool in isolation. Reasons for any reported decline in the coverage of protective care practices for unattended births need to be understood and addressed separately for each context. This requires a deeper understanding of the underlying causes of local trends beyond shifts in birth attendance, such as increased coverage of community-based interventions (e.g. local health education programmes and antenatal care), secular improvements due to increased maternal education and household income over the study periods, and potentially overcrowding and lack of capacity in institutions as a result of increasing numbers.

There is still much room for improvement in the provision of essential newborn care in South Asia and the global health community is extremely active in running trials that try to address this gap. However, there is perhaps a missed opportunity here. Control arm data can provide individual level data on provision of newborn care practices in *non-intervention* areas (which are more relevant to policy-makers dealing with the immediate challenges of the general population) with a level of detail that is rarely available in a non-research context. Applying the methods demonstrated here, considerable value over and above the trial-specific questions could be achieved by using the control arm data to understand recent trends in newborn care practice within the context of the local health environment. To capitalise on this information, those responsible for organising and delivering care need to have ready access to the insights generated, and new data sharing requirements can make an important contribution to this [31].

## Supporting Information

S1 File Dataset for Eastern India. (XLSX)

S2 File Dataset for Bangladesh. (XLSX)

S3 File Dataset for Dhanusha (Nepal). (XLSX)

S4 File Dataset for Makwanpur (Nepal). (XLSX)

S1 Table Brief description of each trial area. This table has been reproduced from reference [15].
(DOCX)

S2 Table Relevant questions used for birth practice variables. For multiple choice answers, the answers that would give a “Yes” to the birth practice are highlighted. This table has been reproduced from reference [15].
(DOCX)

S3 Table Birth practices included in the study. An “X” indicates whether relevant information about the birth practice is available for that study. This table has been reproduced from reference [15].
(DOCX)

S4 Table Definitions used for each delivery type in each study area. This table has been reproduced from reference [15].
(DOCX)
