# Supplementary material for: Generating Insights from Trends in Newborn Care Practices from Prospective Population-Based Studies: Examples from India, Bangladesh and Nepal
Source: PLoS One. 2015 Jul 15;10(7):e0127893. doi: 10.1371/journal.pone.0127893 (PMC4503724; doi:10.1371/journal.pone.0127893)
Supplement: S1 Table — This table has been reproduced from reference [15]. (DOCX) [file pone.0127893.s005.docx]

**S1 Table - Brief description of each trial area.** This table has been reproduced from reference [15].

| **Project partner**  Study setting | **Perinatal Care Project (PCP)**  Rural Bangladesh | **Ekjut**  Rural eastern India | **MIRA (Makwanpur)**  Rural Nepal (middle hills) | **MIRA (Dhanusha)**  Rural Nepal (lowlands) |
| --- | --- | --- | --- | --- |
| Location of clusters | Three districts: Bogra, Maulvibazaar and Faridpur | Three districts of Jharkhand and Odisha: Keonjhar, West Singhbhum and Saraikela | Makwanpur district, central region middle hills. | Dhanusha district, plains of Nepal. |
| Period for which data are included (dates of birth) | 1^st^ Feb 2005 - 31^st^ Dec 2009 | 1^st^ July 2005 - 30^th^ June 2008 | 1^st^ November 2001 - 31^st^ October 2004 (Phase 1)  1^st^ November 2004 – 31^st^ October 2008 (Phase 2) | 1^st^ September 2006 - 13^th^ April 2011 |
| Total estimated population in the areas studies were conducted in | 478 000 | 228 000 | 400 000 | 670 000 |
| Design | Two-by-two factorial cluster RCT which ran from 1^st^ Feb 2005 to 31^st^ Dec 2007. A new trial took place using the same clusters from 2009-2011. Data continued to be collected in all clusters from 1^st^ Jan 2008 to 31 Dec 2009 and continued into the new trial period (not included here). | Cluster RCT | Cluster RCT | Two-by-two factorial cluster RCT |
| Stratification | By district (3 strata) | By district (3 strata) | None | By cluster size (2 strata) |
| Cluster characteristics | Villages making up a union | 8-10 villages with most residents classified as tribal or Other Backward Class | Village development committee | Village development committee |
| Total number of clusters  (Number included in this study) | 18 (9) | 36 (18) | 24 (12) (Phase 1)  30 (6) (Phase 2 – former control clusters became intervention clusters and 6 new control clusters recruited) | 60 (30) |
| Annual births sampled per cluster (after exclusions):  Mean (SD) | 596 (119) | 171 (38) | 115 (70) | 104 (17) |
| Approximate cluster population | 28 000 | 6400 | 4000 | 8000 |
