# Supplementary material for: Generating Insights from Trends in Newborn Care Practices from Prospective Population-Based Studies: Examples from India, Bangladesh and Nepal
Source: PLoS One. 2015 Jul 15;10(7):e0127893. doi: 10.1371/journal.pone.0127893 (PMC4503724; doi:10.1371/journal.pone.0127893)
Supplement: S3 Table — An “X” indicates whether relevant information about the birth practice is available for that study. This table has been reproduced from reference [15]. (DOCX) [file pone.0127893.s007.docx]

**S3 Table – Birth practices included in the study.** An “X” indicates whether relevant information about the birth practice is available for that study. This table has been reproduced from reference [15].

| **Birth** **Practice** | **Eastern India** | **Bangladesh** | **Makwanpur (Nepal)** | **Dhanusha (Nepal)** |
| --- | --- | --- | --- | --- |
| **Intrapartum hygienic care practices** | | | | |
| Attendant washed hands before delivery | X | X | X | X |
| Clean Delivery Kit used | X | X | X | X |
| Attendant used disposable gloves | X | X |  |  |
| Plastic sheet used | X | X |  |  |
| **Intrapartum and postnatal cord care** | | | | |
| Thread / clamp used during delivery | X | X | X (Phase 2) | X |
| Cord tied with boiled thread | X | X |  |  |
| Cord cut with new/sterile blade | X | X | X^†^ | X^†^ |
| Cord care (nothing or only antiseptic applied to cord stump) | X | X | X^*^ | X^*^ |
| **Postnatal newborn care** | | | | |
| Immediate wiping (within 10 minutes) | X | X |  |  |
| Clean cloth used for wrapping | X | X |  |  |
| Immediate warmth^‡^ (wrapping or skin-to-skin contact within 10 minutes) | X | X | X^**^ | X^**^ |
| Skin-to-skin contact between mother and baby within 30 minutes | X | X |  |  |
| Immediate breastfeeding (within 1 hour) | X | X | X | X |
| Colostrum not discarded |  |  | X | X |
| No pre-lacteal feed (breast milk first food) | X | X | X | X |
| Delayed bathing (baby not bathed for at least 6 hours) | X | X | X | X^‡^ |
| Only breast milk in first 24 hours | X | X |  |  |

* In both Nepal sites, the available data was on whether nothing was applied to the cord or ‘medicine/dettol’.

† For eastern India and Bangladesh, there was data both on whether a new blade was bought for cord cutting and whether the implement used for cutting (whether a new blade or not) had been boiled prior to use. For the Nepal sites, there was not information on whether the blade was new and only whether the cord had been cut with a boiled blade.

‡ For Dhanusha detailed timing information was not available and so delayed bathing was defined as “not bathed in 24 hours”.

** For eastern India and Bangladesh, thermal care was defined as “either wrapped or skin-to-skin contact within 10 minutes”. Questions on skin-to-skin were not asked in Nepal and so thermal care was defined as wrapping “Immediately” (Dhanusha) or “within 10 minutes” (Makwanpur).
