# Supplementary material for: Generating Insights from Trends in Newborn Care Practices from Prospective Population-Based Studies: Examples from India, Bangladesh and Nepal
Source: PLoS One. 2015 Jul 15;10(7):e0127893. doi: 10.1371/journal.pone.0127893 (PMC4503724; doi:10.1371/journal.pone.0127893)
Supplement: S4 Table — This table has been reproduced from reference [15]. (DOCX) [file pone.0127893.s008.docx]

**S4 Table - Definitions used for each delivery type in each study area.** This table has been reproduced from reference [15].

| **Delivery Type** | **Definitions used in this study** | | | |
| --- | --- | --- | --- | --- |
|  | East India | Bangladesh | Nepal, Makwanpur | Nepal,  Dhanusha |
| Institution | Government hospital; charitable hospital; NGO facility; private facility. | Medical college hospital; district hospital; maternal and child welfare centre; upazilla health complex; union health and family welfare centre; NGO facility; Private facility; other hospital | Hospital; private hospital; primary health care; health post. | Janakpur or other hospital; NGO facility; Private facility; other hospital; Government health facility. |
| Home Skilled Birth Attendant (SBA) | Not in an institution and main attendant one of: Doctor or Nurse | Not in an institution and main attendant one of: Doctor; family welfare visitor; family welfare assistant; SBA; other outreach worker; midwife. | Not in an institution and main attendant one of: Doctor, Nurse, Auxiliary Nurse Midwife | Not in an institution and main attendant one of: Doctor, Nurse or Auxiliary Nurse Midwife |
| Home non-SBA | Not in an institution and not an SBA. Includes self, family, village doctor and traditional birth attendants. | Not in an institution and not an SBA. Includes self, family, village doctor and traditional birth attendants. | Not in an institution and not an SBA. Includes self, family and traditional birth attendants. | Not in an institution and not an SBA. Includes self, family, village doctor and traditional birth attendants. |
